# Nonirritant and Cytocompatible *Tinospora cordifolia* Nanoparticles for Topical Antioxidant Treatments

**DOI:** 10.1155/2020/3637098

**Published:** 2020-08-21

**Authors:** Jeimmy González-Masís, Jorge M. Cubero-Sesin, Yendry Regina Corrales-Ureña, Sara González-Camacho, Nohelia Mora-Ugalde, José Roberto Vega-Baudrit, Klaus Rischka, Virendra Verma, Rodolfo J. Gonzalez-Paz

**Affiliations:** ^1^Centro de Investigación y Extensión en Materiales, Escuela de Ciencia e Ingeniería de Los Materiales, Instituto Tecnológico de Costa Rica, Cartago 159-7050, Costa Rica; ^2^National Laboratory of Nanotechnology LANOTEC, National Center of High Technology (CeNAT-CONARE), Pavas 1174-1200, San José, Costa Rica; ^3^Biological Assay Laboratory (LEBi), Universidad de Costa Rica, San José 11501-2060, Costa Rica; ^4^National Center for Biotechnological Innovations (CENIBiot), National Center of High Technology (-CeNAT-CONARE), Pavas 1174-1200, San José, Costa Rica; ^5^Laboratorio de Polímeros (POLIUNA), Universidad Nacional, Heredia 86-3000, Costa Rica; ^6^Fraunhofer Institute for Manufacturing Technology and Advanced Materials, Adhesive Bonding Technology and Surfaces, Wiener Straße 12, 28359, Bremen, Germany; ^7^Ayurvedic Physician Private Clinic & Panchakarma Centre, Varanasi, India

## Abstract

*Tinospora cordifolia* extract contains antioxidants such as polyphenols, and thus, it has been used as a natural phytochemical antioxidant therapeutic agent. Many of these compounds are insoluble or only partially soluble in water. In this study, we produced a novel aqueous nanoparticle formulation, with an average particle size of 182.9 ± 3.8 nm, to improve the dispersion of the bioactive compounds in water and to increment its bioavailability. The nanoparticles are composed of polyphenols, alkaloids, and glycosides. We studied the effect of this nanoparticle formulation on mouse 3T3 fibroblast cell viability and New Zealand rabbit dermal irritability tests. Concentrations of 2.5, 25, and 250 *µ*g/mL resulted in similar cell viability to cells in culture media. An intermediate concentration of 12.45 mg/ml was used for the acute dermal irritability test. There were no severe alterations that compromised animal health. These results represent a precedent for application of such nanoparticles derived from plant stems, such as *Tinospora cordifolia*, in biomedicine and in antiaging cosmetic treatments.

## 1. Introduction

*Tinospora cordifolia*, also known as Guduchi, is a deciduous climbing shrub, found in India, especially in tropical areas, being famous for its numerous properties and uses in the ancient Ayurveda and Tibetan medicinal system [1]. *Tinospora cordifolia* is found ascending to an altitude of 1000 feet in Southeast Asian countries such as Indonesia, the Philippines, Thailand, Myanmar, China, and Sri Lanka [2]. It is used to prepare tonics, vitalizers, antiallergics, and treatments for diabetes mellitus, among others [3, 4]. The beneficial properties of this plant are associated with a large number of chemical components, such as alkaloids, diterpenoid lactones, glycosides, steroids, sesquiterpenoids, phenolic compounds, and aliphatic compounds that present immunomodulatory activities [5]. These compounds exist in plant stem, leaves, roots, and fruits [4]. Several studies have reported that *Tinospora cordifolia* ethanolic extract (TEC) exhibits higher antioxidant activity, using *in vitro* antioxidant activity screening models such as nitric oxide and superoxide radical scavenging activity, inhibition of lipid peroxidation, and reduction of ferric ions [6]. This antioxidant properties are attributed to the strong free radical scavenging properties against superoxide anion (O^2−^) and hydroxyl radicals (OH), through a series of coupled reactions with the help of enzymes [7, 8]. In regards to toxicity and side effects, the Ayurvedic literature reports that *Tinospora* can cause constipation, if it is taken regularly at high doses. However, it has been reported that no side effects or adverse reactions have been observed when the stem extract of *Tinospora* was administered in rabbits up to an oral dose as high as 1.6 g/kg and to rats up to a dose of 1 g/kg of total plant extract. Little is known about the toxicology of this product in humans [9]. The presence of compounds such as 2,4-bis(1,1-dimethyl-ethyl)-phenol and palmitic acid could provide the antioxidant properties [10]. Many of these compounds are partially soluble in water, and this limits its use in aqueous formulations. The usual preparation protocol is to cut part of the plant into small pieces, let to dry (usually in the shade), and grind these pieces to produce a powder, which can be dispersed in water-ethanol or methanol mixtures [1, 3, 9]. To improve the solubility in water and increase the bioavailability of the bioactive compounds, we will produce a nanosuspension of TEC in water applying sonication and filtration to decrease the aggregate sizes of insoluble compounds. Characterization techniques such as using Fourier-transform infrared spectroscopy (FTIR), gas chromatography-mass spectrometry (GC-MS), and ^13^C- and ^1^H-NMR (nuclear resonance spectroscopy) were used. The nanoparticle suspensions will be characterized using dynamic light scattering (DLS) and atomic force microscopy (AFM) to determine their particle size and morphology. The effect of the *Tinospora cordifolia* nanoparticle suspension on 3T3 fibroblast viability and animal dermal irritability was also evaluated.

## 2. Experimental

### 2.1. Production of Nanoparticle Aqueous Formulation

Dried *Tinospora cordifolia* stems from Varanasi (latitude: 25°19′3.52″N and longitude: 82°58′26.09), India, were pulverized using an industrial blender and mixed with 250 mL of ethanol. The alcoholic solution, with a concentration of 104.7 mg/mL, was filtered and placed in an oven at 45°C for 16 h to obtain a dry extract. A 13.2 mg/mL aqueous suspension was prepared in ultrapurified water and sonicated to obtain the nanoparticulate solution. The conditions of the ultrasonic treatment were 4 W and 40% amplitude for 5 min, using a probe of 6 mm diameter. The solution was filtered with a sterile filter of 0.2 *µ*m (Sartorius, Germany) and diluted to 2.5 *μ*g/mL, 25 *μ*g/mL, and 250 *μ*g/mL concentrations using ultrapure water.

### 2.2. Characterization of the *Tinospora cordifolia* Extract and Nanoparticle Suspension

#### 2.2.1. Fourier-Transform Infrared Spectroscopy (FTIR)

A Nicolet 6700 spectrophotometer was used, encompassing 4000 to 400 cm^−1^ wavenumber with a standard resolution of 0.09 cm^−1^ and a scanning speed of 32 spectrums/s.

#### 2.2.2. Matrix-Assisted Laser Desorption/Ionization Time-of-Flight Mass Spectrometry (MALDI-ToF MS)

Spectra were recorded on a Bruker Autoflex Speed (Bruker Daltonics, Bremen, Germany) mass spectrometer. The applied matrix was 2,5-dihydroxybenzoic acid (DHB, Sigma Aldrich, Germany) in methanol. The sample was dissolved in methanol. The matrix and sample were mixed in the ratio 10 : 1 (matrix : sample) and applied on a stainless steel target plate. The mixture was dried in the open air (dried droplet). A calibration was applied by using PEG 400. The spectrum was recorded in the positive reflection mode by the accumulation of 5 × 1000 scans.

#### 2.2.3. Nuclear Magnetic Resonance Spectroscopy (NMR)

NMR spectra were recorded in CDCl_3_ as solvent, with a Bruker Advance DRX-600 spectrometer equipped with a Prodigy BBIO cryoprobe (^1^H at 600.22 MHz; ^13^C at 150.94 MHz). ^1^H shifts were calibrated by using the chemical shifts of the CDCl_3_ solvent signal (*δ H* = 7.26 ppm, 300K) as an internal standard [11]. ^13^C chemical shifts were calibrated using the IUPAC recommendations applying the conversion factor of 0.25144953 for the calculation of the absolute frequency of ^13^C (*δ* = 0.0 ppm) [12].

#### 2.2.4. Total Polyphenol Content

Solutions with a concentration of 100, 50, 10, 5, and 1 *µ*g/mL were prepared for the determination of the calibration curve. 0.75 mL of distilled water, 0.5 mL of the solutions, and 0.625 mL of the Folin reagent (1N) were placed in a volumetric flask of 5 mL. Then, the flask was filled with a solution of Na_2_CO_3_·10H_2_O (20% m/v). The mixtures were incubated for 40 min at room temperature in the dark. After the reaction period, the absorbance of the solutions was measured at a wavelength of 725 nm in a Shimadzu UV-Vis spectrophotometer (model UV-1800, Shimadzu Corporation, Kyoto, Japan).

#### 2.2.5. Dynamic Light Scattering (DLS)

The average nanoparticle hydrodynamic diameter was measured using a Malvern Zetasizer instrument at 25°C.

#### 2.2.6. Atomic Force Microscopy (AFM)

The 20 *μ*L nanoparticle solution was placed on a freshly cleaved mica substrate and dried at room environmental conditions overnight. The sample topography was analyzed in air using an atomic force microscope (Asylum Research, Santa Barbara, CA), operated in the tapping mode. Silicon probes (model Tap150Al-G), backside with a resonance frequency of 150 kHz and a force constant of 5 N/m, were used.

#### 2.2.7. Cell Culture

Mouse 3T3 fibroblast cell line (ATCC, USA) was incubated (Binder, Germany) and cultured in standard conditions (37°C, 5% CO_2_) in Dulbecco's modified Eagle's medium (DMEM, Gibco®). The medium was supplemented with 10% v/v fetal bovine serum (FBS) (Sigma-Aldrich) and changed every other day. The cells were subcultured using routine trypsin/EDTA (Merck) procedures.

#### 2.2.8. Viability Assay

Exponentially growing 3T3 cells were seeded in a 96-well plate at a density of 700 cells/100 *µ*L/well and left in the incubator growing for 24 h. Afterwards, 50 *µ*l of the *Tinospora cordifolia* nanoparticle suspensions of 2.5, 25, and 250 *µ*g/mL, containing resazurin salts (Sigma-Aldrich), was added to the culture with or without cells. Fluorescence readings were carried out using a microplate reader (Synergy H1 Hybrid, Biotek) with a gain of 70 at 0, 24, 48, and 72 h, and excitation and emission wavelengths of 540 and 590 nm, respectively. The fluorescence blank was subtracted from the average of the data (supernatant with the culture medium in the absence of the cells), and the percentage of viability was calculated using the following equation:

(1) $$\begin{array}{c} \text{viability }\left(\%\right)=\frac{\text{average number of cells with treatment}}{\text{average number of cells without treatment}}\times 100. \end{array}$$

#### 2.2.9. Acute Dermal Irritability Test

The test evaluated the irritability of the aqueous solution of *Tinospora cordifolia* nanoparticle suspension at a concentration of 12.45 mg/mL and pH of 7, on the healthy skin of a New Zealand rabbit.

- Administration of the substance to be tested: at least 24 h before the test, the hair of the animals was carefully cut on both sides of the back, in an approximate area of 6 cm^2^. Only animals whose skin was intact were used. A single dose of 0.5 mL of the undiluted test substance was applied with a 4-hour exposure, after which the residual material was removed using distilled water, so as not to disturb the integrity of the epidermis.
- Observation: inspections of the skin (erythema and edema) of the live animals were conducted at 24 h, 48 h, and 72 h after the administration of the test substance. Daily observation of the animals was conducted for 14 days after the application to determine the health status and subsequent responses or recovery. Weekends were omitted once it was demonstrated that after 72 hours of application, any signs of irritation or corrosion had disappeared.
- Irritation evaluation method: the records of dermal irritation were evaluated in conjunction with the nature and reversibility of the observed response. The severity scores were assigned through the use of the Primary Skin Irritation Scale. The individual scores did not constitute an absolute norm for the irritating properties of a material. They were considered as reference values and were supported by a complete description and evaluation of the observations.
- Classification of the substance to be tested: at the end of the observation period, the substance was classified according to the parameters given by the Globally Harmonized System of Classification and Labeling of Chemicals (GHS) and the Organization for Economic Cooperation and Development (OECD), as shown in Tables 1 and 2. The parameters to evaluate the animal health and the scale are shown in Tables 3 and 4, and the scale for evaluation of erythema and edema reactions is shown in Table 5.

## 3. Results and Discussion

**Figure 1:**
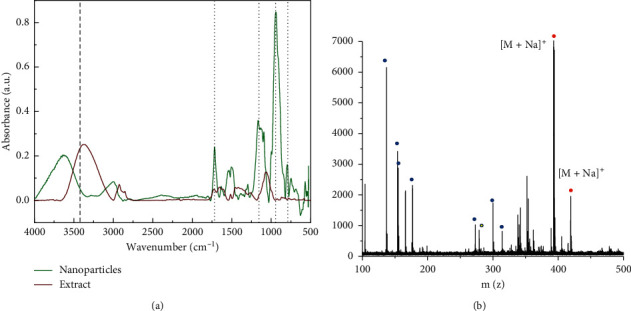
(a) FTIR spectra of the TEC extract and TEC-NP aqueous suspension. (b) MALDI-TOF spectrum in the range of 100 to 500 Da of the TEC-NP dried suspension.

The chemical structure of the natural extract (TEC) and the nanoparticle suspensions (TEC-NPs) was analyzed using FTIR, as shown in Figure 1(a). The TEC spectrum shows a peak at 3380 cm^−1^ and 1080 cm^−1^ from the hydroxyl group, corresponding to free alcohols and/or water. The peak at 3395 cm^−1^ could be associated with phenolic compounds [9, 13]. The presence of hydroxyl groups could be associated with compounds such as polyphenols, fatty acids, and monosaccharides [13, 14]. The peak at 1730 cm^−1^ is associated with the stretching vibration of the C=O steroids [9, 14, 15]. The peak at 1630 cm^−1^ corresponds to the C=C bond of olefin. Peaks at 1180 cm^−1^ and 950 cm^−1^, both present in the nanoparticles and the extract, could be associated with the stretching vibrations of the C=O and C-O-C groups, respectively [15]. Also, near 720 cm^−1^, a strong band appears, typical of the C-H groups [1, 16]. Comparison of the bands of the extract and the nanoparticles reveals peak shifts at higher wavenumbers, as well as an increase in the intensity of the absorption bands. This result suggests that some compounds could be less soluble in water than in ethanol, and their aggregates were removed during filtration. Also, the difference in chemical environment that influences the hydrogen bond formation and electrostatic interactions produce shifts on the absorption bands [17].

**Figure 2:**
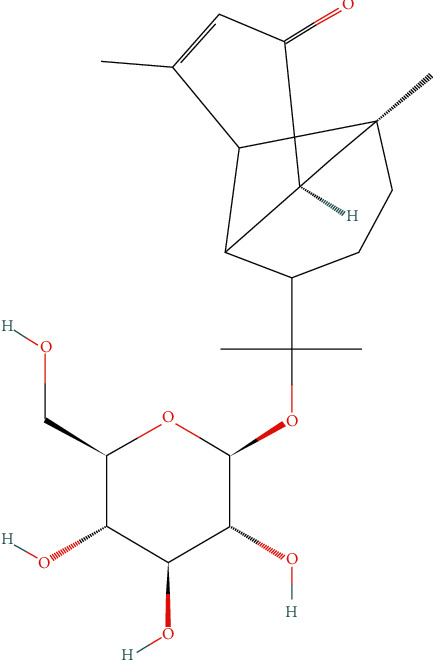
Tinocordiside compound 2D chemical structure [22].

Figure 1(b) shows the peaks at *m*/*z* = 393.122 and *m*/*z* = 419.207, indicated by red dots, which are associated with compounds contained in the extract such as secondary metabolites and polyphenol oligomers [18–20]. The compound at *m*/*z* = 419.207 corresponds to sodium adduct of tinocordiside, a tricyclic sesquiterpenoid glycoside [21]. A 2D model of the structure is shown in Figure 2.

Tinocordiside is a glycoside well known for its traditional uses in treatment of neurological disorders such as ALS, Parkinson's disease, dementia, motor and cognitive deficits, and neuron loss in the spine and hypothalamus [23]. Immunomodulatory activity is also attributed to this compound [24] and for thrombolytic drugs [25]. The blue dots correspond to the DHB signal at *m*/*z* = 136.994, 154.004, 155.012, 177.004, 273.030, 300.150, and 361.976. The yellow dot at *m*/*z* = 279.086 corresponds to triphenylphoshinoxide + H^+^ that is correlated to contamination from the packaging container where the extract was stored.

**Figure 3:**
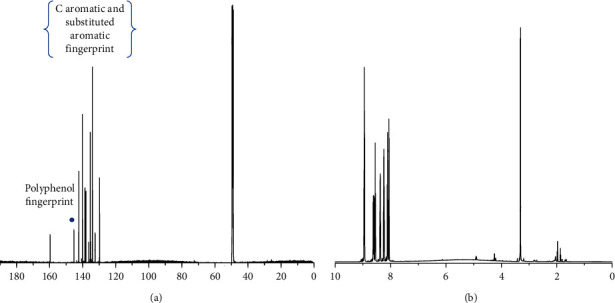
(a) ^13^C-NMR and (b) ^1^H-NMR of the TEC-NP suspension dried and redissolved in CDCl_3_.

The ^13^C-NMR spectral data in Figure 3(a) show C aromatic atoms at 121.3, 124.3, 127.8, 129.5, 130.16, 131.67, 133.8, 136.7, and 136.8 ppm, which correspond to C-H shifts from aromatic rings and are the fingerprint of alkaloids such as N-formylannonaine [26]. The C polyphenol fingerprint is 140.1, 142, and 145 ppm [27].

The ^1^H-NMR spectral data in Figure 3(b) show protons at 1.97 ppm assigned to CH_2_ groups, polysaccharides, or aromatic OH groups from polyphenols at 4.91 ppm, and the alkaloid fingerprint between 8 and 9 ppm [28]. Carbon ^13^C-NMR and ^1^H-NMR results confirm that preferential presence of polyphenols. The phenols in the extract were quantified using the Folin–Ciocalteu test. The results are shown in Table 6. The Folin–Ciocalteu reagent is sensitive to the reduction of hydroxyl groups of reducing sugars, and the values should be only taken as an approximation.

**Figure 4:**
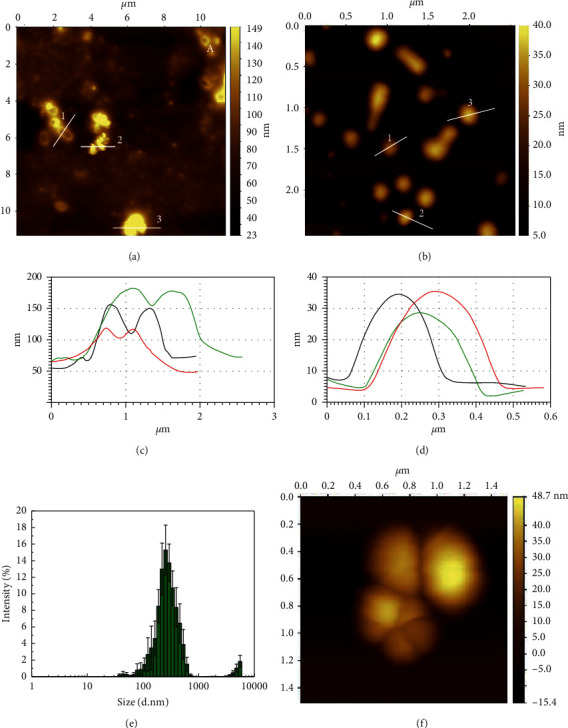
AFM topography image of (a) alcoholic TEC extract, (b) TEC NPs, (c, d) profile cross section (P) of lines shown in (a) and (b), respectively, (e) DLS size distribution, and (f) AFM topography image of NP aggregate.

The nanoparticle formation was confirmed using AFM.  Figure 4(a) shows aggregates contained in the filtered ethanolic TEC extract with a height above 100 nm. The profiles of the aggregates show particles with heights between 60 and 150 nm and widths between 500 nm and 2 *µ*m (Figure 4(c)). These aggregates could be correlated to the partially soluble and insoluble compounds previously described.  Figure 4(b) shows nanoparticles with heights of 20–30 nm and an average of 560 ± 130 nm diameter, confirming the effectiveness of sonication and filtration processes in order to decrease the aggregate size and to form micellar-like structures, Figure 4(d). However, according to the DLS size distribution analysis, the sample is polydisperse (Figure 4(e)).  Figure 4(f) shows aggregates of particles that tend to form on the mica surface, and these results suggest that aggregates could form in the solution and an extra cosurfactant could be added to stabilize the nanosuspension.

### 3.1. Nanoparticle Cytotoxicity and Irritability Tests

**Figure 5:**
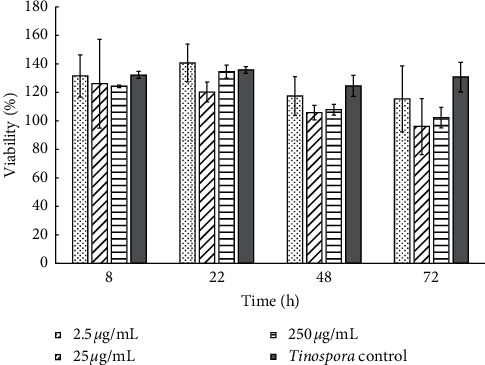
Cellular viability of 3T3 cell line incubated at different times of TEC-NPs in three concentrations (mean% ± SD).

Significance difference in viability between the samples and the control was determined by an analysis of variance (ANOVA) test, followed by a Tukey post hoc test. No significant differences at *p* < 0.05 between the control of viability (100% growth) and the TEC-NP solutions were found. All aqueous solutions of nanoparticles are not cytotoxic to the 3T3 cell line used, achieving an increase higher than the control (100%), as shown in Figure 5. The maximum value is obtained from the aqueous solution of nanoparticles with a concentration of 2.5 *μ*g/mL, after 22 hours, whose viability is slightly higher than 140%.

As observed in the summary of Tables 7 and 8, there were no severe alterations that compromised animal health and welfare. There was also no obvious irritation of the test substance in contact with the skin. Therefore, the TEC-NP in the concentration 12.45 mg/mL is classified as nonirritating, since no evident irritation was observed during the entire period of the test. These results are consistent with the research of other authors, without obtaining adverse results [4, 29].

## 4. Conclusions

Stable *Tinospora cordifolia* stem derivate nanoparticles, with a hydrodynamic diameter of 182.9 ± 3.8 nm, were obtained in water by sonication. Polyphenols, alkaloids, and tinocordiside compose the nanoparticles. The nanoparticle cellular viability tests showed that they do not decrease the 3T3 fibroblast viability at a concentration range of 2.5–250 *μ*g/mL and that are cytocompatible. In addition, its effect in dermal irritability showed no skin irritation of the New Zealand rabbit at 12.45 mg/mL. In vivo tests could confirm the viability in humans and a potential application in biomedicine, antiageing formulations, or others.

## Figures and Tables

**Table 1:** Substance classification tested according to GHS/OECD parameters.

Corrosive (category 1)	Corrosive potential subclassification	Corrosive in 1 or more animals	
		Exposition	Observation
Corrosive	Corrosive subcategory 1A	≤3 minutes	≤1 hour
	Corrosive subcategory 1B	≤3 minutes to ≤1 hour	≤14 days
	Corrosive subcategory 1C	> 1 hour to ≤4 hours	≤14 days

**Table 2:** Substance classification criterion tested according to GHS/OECD parameters.

Classification	Criterion
Irritative (Category 2)	(1) Average of ≥2.3 ≤ 4.0 for erythema/eschar or for edema in at least 2 of the 3 animals tested at 24 hours, 48 hours, and 72 hours after removing the patch, or if the reactions are delayed, for classifications in 3 consecutive days after the appearance of skin reactions, or (2) inflammation that persists at the end of the observation period, normally 14 days and in at least 2 animals, particularly taking into account alopecia (defined area) hyperkeratosis, hyperplasia, and flaking, or (3) in some cases when there is a pronounced response variability between the animals, with well-defined positive effects related to the exposure chemical in an animal but less than the previous criteria

Medium irritant (Category 3)	Average of ≥1.5 < 2.3 for erythema/eschar or for edema in at least 2 of the 3 animals tested at 24 hours, 48 hours, and 72 hours or, if the reactions are delayed, for ratings on 3 consecutive days after the onset of skin reactions (when they are not included in the previous category of irritation)

Not irritating	When the criteria of Categories 1–3 are not met

**Table 3:** Parameters of animal health evaluation.

Parameter	Description
Hair appearance	Texture, color, fall
Skin appearance	Redness, dryness, exudation
Eyes and mucous membranes	Redness, dryness, abnormal secretion
Ataxia	Loss of balance, erratic walk
Paralysis	Loss of response in any limb
Reaction to stimuli	Response to touch or noise
Peripheral vasoconstriction	Pallor
Peripheral vasodilation	Redness
Piloerection	Stiff hair
Salivation	Excess of buccal secretion
Motor activity	Increase or decrease in normal activity, reflect or not
Tremors and convulsions	Spontaneous abnormal muscle contraction, contraction or uncontrolled muscular stretching
Breathing	Increase or decrease in respiratory rate
Dehydration	Robin's test: pinching the skin, without returning to its normal position
Diarrhea	Soft stools or watery stool
Lose	Weight lose

**Table 4:** Scale of values for animal health assessment parameters.

Grade	Description
0	There is no alteration
1	Mild alteration that does not affect the other parameters
2	Moderate alteration that does not affect the general behavior of the animal
3	Severe alteration that affects the general behavior of the animal

**Table 5:** Evaluation scale of dermal reactions (erythema and edema).

Erythema formation	Value	Edema formation	Value
No presence of erythema	0	No edema formation	0
Very slight erythema (almost imperceptible)	1	Very slight edema (almost imperceptible)	1
Well-defined erythema	2	Light edema (well-defined area with significant elevation)	2
Moderate-to-severe erythema	3	Moderate edema (elevation of approximately 1 mm)	3
Severe erythema (red beet) with formation of scabs (deep lesions)	4	Severe edema (elevation greater than 1 mm and extending beyond the exposure area)	4
Maximum possible	4	Maximum possible	4

**Table 6:** Phenol quantification by the Folin–Ciocalteu method for *Tinospora cordifolia* ethanolic extract and nanoparticles.

Sample	Concentration (mg/mL)	
	Mean	Standard deviation
Alcoholic extract	0.94	0.02
Nanoparticles	0.09	0.01

**Table 7:** Evaluation scale of dermal reactions (erythema and edema).

Erythema	Day 1			Day 2			Day 3			Day 4			Day 5			Day 6			Day 7		
Animal	1	2	3	1	2	3	1	2	3	1	2	3	1	2	3	1	2	3	1	2	3
Hair appearance	0	0	0	0	0	0	0	0	0	0	0	0	0	0	0	0	0	0	0	0	0
Skin appearance	0	0	0	0	0	0	0	0	0	0	0	0	0	0	0	0	0	0	0	0	0
Eyes and mucous membranes	0	0	0	0	0	0	0	0	0	0	0	0	0	0	0	0	0	0	0	0	0
Ataxia	0	0	0	0	0	0	0	0	0	0	0	0	0	0	0	0	0	0	0	0	0
Paralysis	0	0	0	0	0	0	0	0	0	0	0	0	0	0	0	0	0	0	0	0	0
Reaction to stimuli	0	0	0	0	0	0	0	0	0	0	0	0	0	0	0	0	0	0	0	0	0
Peripheral vasoconstriction	0	0	0	0	0	0	0	0	0	0	0	0	0	0	0	0	0	0	0	0	0
Peripheral vasodilation	0	0	0	0	0	0	0	0	0	0	0	0	0	0	0	0	0	0	0	0	0
Piloerection	0	0	0	0	0	0	0	0	0	0	0	0	0	0	0	0	0	0	0	0	0
Salivation	0	0	0	0	0	0	0	0	0	0	0	0	0	0	0	0	0	0	0	0	0
Motor activity	0	0	0	0	0	0	0	0	0	0	0	0	0	0	0	0	0	0	0	0	0
Tremors and convulsions	0	0	0	0	0	0	0	0	0	0	0	0	0	0	0	0	0	0	0	0	0
Breathing	0	0	0	0	0	0	0	0	0	0	0	0	0	0	0	0	0	0	0	0	0
Dehydration	0	0	0	0	0	0	0	0	0	0	0	0	0	0	0	0	0	0	0	0	0
Diarrhea	0	0	0	0	0	0	0	0	0	0	0	0	0	0	0	0	0	0	0	0	0

**Table 8:** Evaluation scale of dermal reactions of edema.

Edema	Day 8			Day 9			Day 10			Day 11			Day 12			Day 13			Day 14		
Animal	1	2	3	1	2	3	1	2	3	1	2	3	1	2	3	1	2	3	1	2	3
Hair appearance	0	0	0	0	0	0	0	0	0	0	0	0	0	0	0	0	0	0	0	0	0
Skin appearance	0	0	0	0	0	0	0	0	0	0	0	0	0	0	0	0	0	0	0	0	0
Eyes and mucous membranes	0	0	0	0	0	0	0	0	0	0	0	0	0	0	0	0	0	0	0	0	0
Ataxia	0	0	0	0	0	0	0	0	0	0	0	0	0	0	0	0	0	0	0	0	0
Paralysis	0	0	0	0	0	0	0	0	0	0	0	0	0	0	0	0	0	0	0	0	0
Reaction to stimuli	0	0	0	0	0	0	0	0	0	0	0	0	0	0	0	0	0	0	0	0	0
Peripheral vasoconstriction	0	0	0	0	0	0	0	0	0	0	0	0	0	0	0	0	0	0	0	0	0
Peripheral vasodilation	0	0	0	0	0	0	0	0	0	0	0	0	0	0	0	0	0	0	0	0	0
Piloerection	0	0	0	0	0	0	0	0	0	0	0	0	0	0	0	0	0	0	0	0	0
Salivation	0	0	0	0	0	0	0	0	0	0	0	0	0	0	0	0	0	0	0	0	0
Motor activity	0	0	0	0	0	0	0	0	0	0	0	0	0	0	0	0	0	0	0	0	0
Tremors and convulsions	0	0	0	0	0	0	0	0	0	0	0	0	0	0	0	0	0	0	0	0	0
Breathing	0	0	0	0	0	0	0	0	0	0	0	0	0	0	0	0	0	0	0	0	0
Dehydration	0	0	0	0	0	0	0	0	0	0	0	0	0	0	0	0	0	0	0	0	0
Diarrhea	0	0	0	0	0	0	0	0	0	0	0	0	0	0	0	0	0	0	0	0	0

## Data Availability

The results used to support the findings of this study are available from the corresponding author upon request (drpazsowarigpa@gmail.com).
